# Biochemical and Structural Properties of Cyanases from *Arabidopsis thaliana* and *Oryza sativa*

**DOI:** 10.1371/journal.pone.0018300

**Published:** 2011-03-31

**Authors:** Dan Qian, Lin Jiang, Lu Lu, Chunhong Wei, Yi Li

**Affiliations:** State Key Laboratory of Protein and Plant Gene Research, Peking-Yale Joint Center for Plant Molecular Genetics and Agrobiotechnology, College of Life Sciences, Peking University, Beijing, People's Republic of China; Institut Pasteur, France

## Abstract

Cyanate is toxic to all organisms. Cyanase converts cyanate to CO_2_ and NH_3_ in a bicarbonate-dependent reaction. The biophysical functions and biochemical characteristics of plant cyanases are poorly studied, although it has been investigated in a variety of proteobacteria, cyanobacteria and fungi. In this study, we characterised plant cyanases from *Arabidopsis thaliana* and *Oryza sativa* (AtCYN and OsCYN). Prokaryotic-expressed AtCYN and OsCYN both showed cyanase activity *in vitro*. Temperature had a similar influence on the activity of both cyanases, but pH had a differential impact on AtCYN and OsCYN activity. Homology modelling provided models of monomers of AtCYN and OsCYN, and a coimmunoprecipitation assay and gel filtration indicated that AtCYN and OsCYN formed homodecamers. The analysis of single-residue mutants of AtCYN indicated that the conserved catalytic residues also contributed to the stability of the homodecamer. KCNO treatment inhibited *Arabidopsis* germination and early seedling growth. Plants containing *AtCYN* or *OsCYN* exhibited resistance to KCNO stress, which demonstrated that one role of cyanases in plants is detoxification. Transcription level of *AtCYN* was higher in the flower than in other organs of *Arabidopsis*. *AtCYN* transcription was not significantly affected by KCNO treatment in *Arabidopsis*, but was induced by salt stress. This research broadens our knowledge on plant detoxification of cyanate via cyanase.

## Introduction

Cyanate is generated by the chemical industry and mining wastewater [1]. It is well established that urea is in equilibrium with ammonium cyanate in solution and can spontaneously transform to cyanate [2], and photo-oxidation of cyanide in solution also produces cyanate [3], [4]. Cyanate is toxic. Isocyanate, the active form of cyanate, reacts with amino and carboxyl groups and carbamoylates amino acids, proteins and other molecules, thereby altering their structure, charge and function [5], [6].

Cyanase (EC 4.2.1.104, also known as cyanate lyase or cyanate hydrolase) catalyses a bicarbonate-dependent reaction decomposing cyanate to ammonia and bicarbonate [7]. Cyanase was first identified in *Escherichia coli* and has been characterised in detail in this bacterium [7], [8], [9], [10], [11], [12], [13]. Subsequently, the enzyme was discovered and characterised in proteobacteria [14], [15], [16], [17], [18], cyanobacteria [19], [20], fungi [21] and plants [22]. The monomer of *E. coli* cyanase is a 17 kDa subunit [11] and the functionally active enzyme is a homodecamer of five dimers [8]. Three catalytic residues (Arg96, Glu99 and Ser122) are conserved in cyanase sequences [14], [21], and the enzyme is competitively inhibited by a number of monovalent anions [10]. Cyanase activity is affected by pH and temperature [12], [21]. Among living organisms, cyanase plays a role in detoxification of cyanate and cyanide [23]. Because the direct products of cyanase action are ammonia and bicarbonate, cyanate is utilised as a nitrogen and carbon source in some organisms [17], [24].

Plant cyanase may play roles in different physiological pathways. In *Medicago truncatula*, the transcription level of the putative cyanase gene is lower in nodules than in roots [25]. In *Suaeda aegyptica,* the putative cyanase is upregulated in the leaf during salt accumulation [26]. However, only the cyanase gene in Arabidopsis has been cloned [22]. Because few studies on plant cyanases have been published, more information is needed to understand the roles of cyanase in plants. This study is focused on cyanases in the model plants *Arabidopsis thaliana* and *Oryza sativa*. The objective was to understand the biochemical and structure properties and physiological roles of cyanases from the two species.

## Results

### Isolation of cDNAs encoding cyanases from Arabidopsis thaliana and Oryza sativa

**Figure 1:**
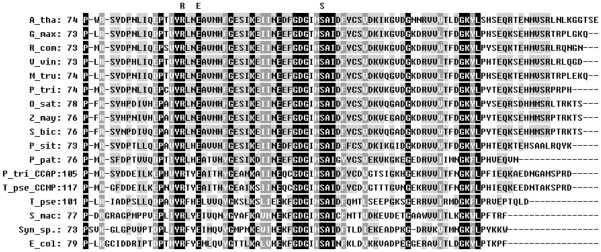
Alignment of catalytic regions of cyanases from fungus, plant and bacterial species. Accession numbers for each cyanase are listed in Table 1. Residues conserved in all sequences are indicated in white type on a black background, the consensus residue or similar residues in all sequences are indicated in white type on a dark grey background and the consensus residue or similar residues in the sequences of cyanases from Dicotyledoneae and Monocotyledoneae are indicated in black type on a light grey background. The predicted catalytic residues in *E. coli* are indicated above the alignment.

To study cyanases in plants, we performed a BLAST-P search for proteins homologous to *E. coli* cyanase in the public NCBI Entrez databases, and located 12 putative plant cyanases in addition to that of *A. thaliana* (Table 1). The multiple amino acid sequence alignments showed that all of the cyanases were highly conserved in the C-terminal region (Figure 1). The plant cyanases shared high sequence identity (35.8%) and similarity (87.4%). The sequences of the six Dicotyledoneae cyanases exhibited 68.4% identity and 94.7% similarity, while the sequences of the three Monocotyledoneae cyanases showed 91.2% identity and 100% similarity. AtCYN and OsCYN shared 70.5% sequence identity and 80.0% similarity. In particular, the three catalytic residues (Arg96, Glu99 and Ser122) in *E. coli* cyanase (EcCYN) were conserved in all putative cyanases from fungi, animals and plants.

**Table 1:** List of cyanases from plants, fungi and bacteria.

Species	Abbreviation	Accession No.	Length	Taxonomic classification
*Arabidopsis thaliana*	*A tha*	NP_188991	168 aa	*Planta; Dicotyledoneae*
*Glycine max*	*G max*	ACU13914	165 aa	*Planta; Dicotyledoneae*
*Ricinus communis*	*R com*	EEF29508	164 aa	*Planta; Dicotyledoneae*
*Vitis vinifera*	*V Vin*	XP_002285393	164 aa	*Planta; Dicotyledoneae*
*Medicago truncatula*	*M tru*	ACJ8540	165 aa	*Planta; Dicotyledoneae*
*Populus trichocarpa*	*P tri*	XP_002315578	162 aa	*Planta; Dicotyledoneae*
*Oryza sativa*	*O sat*	NP_001064827	168 aa	*Planta; Monocotyledoneae*
*Zea mays*	*Z may*	NP_001150815	166 aa	*Planta; Monocotyledoneae*
*Sorghum bicolor*	*S bic*	XP_002467098	166 aa	*Planta; Monocotyledoneae*
*Picea sitchensis*	*P sit*	ABK22377	161 aa	*Planta; Gymnospermae*
*Physcomitrella patens*	*P pat*	XP_001780115	154 aa	*Planta; Bryophyta*
*Phaeodactylum tricornutum* CCAP 1055/1	*P tri CCAP*	XP_002177029	193 aa	*Planta; Bacillariophyta*
*Thalassiosira pseudonana* CCMP1335	*T pse CCMP*	XP_002295609	205 aa	*Planta; Bacillariophyta*
*Trichinella pseudospiralis*	*T pse*	ABR10530	181 aa	*Nematoda*
*Sordaria macrospora*	*S mac*	CAO79555	164 aa	*Fungi*
*Synechocystis* sp.PCC6803	*Syn sp.*	NP_442379	149 aa	*Cyanobacteria*
*Escherichia coli* str. K-12 substr. MG1655	*E col*	NP_414874	156 aa	*Proteobacteria*

**Figure 2:**
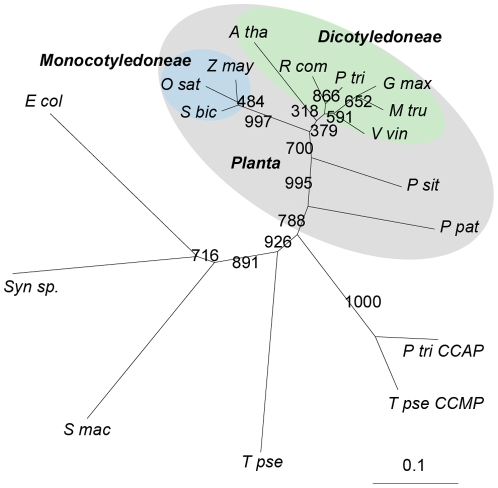
Unrooted tree depicting phylogenetic relationships for catalytic domains of cyanases. The tree was constructed using the neighbour-joining method. Bootstrap values were calculated on 1,000 repeats of the initial alignment using a seed value of 111. The length of lines connecting the proteins indicates the mean number of estimated substitutions per site. And the scale bar represents 0.1 substitution per site. Accession numbers for each cyanase are listed in Table 1. Cyanases from plants are highlighted in grey, cyanases from *Dicotyledoneae* are highlighted in green and cyanases from *Monocotyledoneae* are highlighted in blue.

An unrooted phylogenetic tree representing relationships among these cyanases is presented in Figure 2. The two main clusters represent Dicotyledoneae and Monocotyledoneae cyanases. It indicated that, although the plant cyanase sequences were highly conserved, there has been genetic divergence between dicot and monocot cyanases. Therefore, we cloned the cDNAs of *AtCYN* and *OsCYN*, which encode two 168 aa proteins.

### Effects of pH and temperature on the enzyme activity of heterologously expressed AtCYN and OsCYN

**Figure 3:**
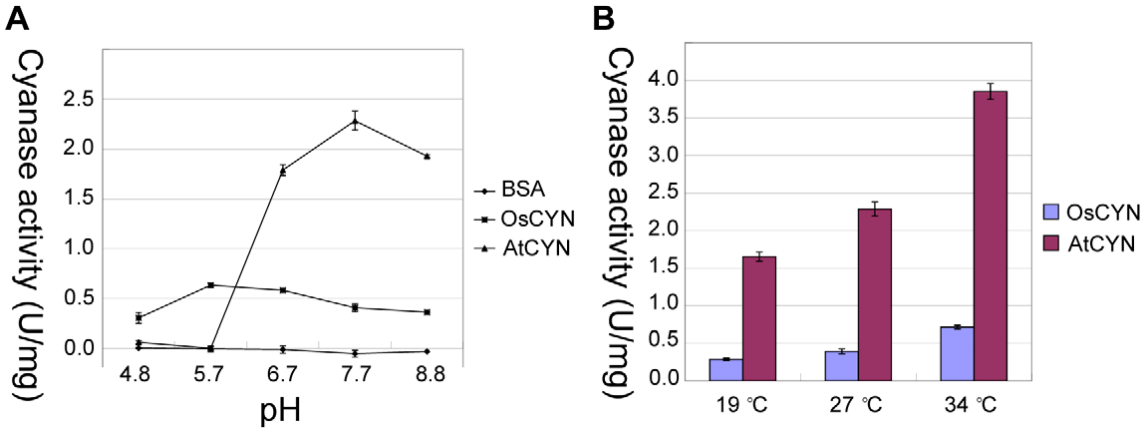
Influence of pH and temperature on cyanase activity. The His:AtCYN and His:OsCYN enzymes were purified and their activities assayed *in vitro*. (A) Effect of pH on cyanase activity at 27°C. BSA was used as a control. (B) Effect of temperature on cyanase activity at pH 7.7.

We produced His-tagged recombinant CYN proteins in *E. coli*. Fortunately, the proteins are soluble and could be purified. The enzyme activities of OsCYN and AtCYN were measured. In the standard assay solution at 27°C, the activity of OsCYN was 0.405 U·mg^–1^ and that of AtCYN was 2.286 U·mg^–1^ (Figure 3A). The biochemical properties of the enzymes were showed in Table 2.

**Table 2:** Biochemical characteristics of heterologously expressed AtCYN and OsCYN.

Kinetic parameter	AtCYN	OsCYN
*Km* NaHCO_3_ (mM)^a^	0.79	0.63
*Km* KCNO (mM)^b^	0.94	7.38
*Vmax* (nmol·mg^−1^·min^−1^)^a^	3820	982
*Vmax* (nmol·mg^−1^·min^−1^)^b^	3980	2080
Optimum pH	7.7	5.7

The apparent *Km* and *Vmax* values were calculated by double-reciprocal plots.

a The cyanase reaction was assayed at 27°C, pH 7.7, in the presence of 5 mM KCNO,

b The cyanase reaction was assayed at 27°C, pH 7.7, in the presence of 5 mM NaHCO_3_.

Activities of the cyanases of *E. coli* and *Sordaria macrospora* are affected by pH and temperature [12], [21]. Therefore, activities of AtCYN and OsCYN were measured across a pH range between 4.8–8.8. As shown in Figure 3A, the optimum pH for AtCYN activity was 7.7 (2.286 U·mg^–1^). At pH 6.7 and 8.8, AtCYN retained >75% activity, but at a low pH (pH 5.7 and 4.8) AtCYN showed no activity. These experiments demonstrated that AtCYN activity is greatly affected by pH. However, OsCYN activity was only slightly affected by pH. The OsCYN activity ranged from 0.303 U·mg^–1^ at pH 4.8 and 0.633 U·mg^–1^ at pH 5.7.

To examine the influence of environmental temperature on cyanase activity, we measured enzyme activity at 19°C, 27°C and 34°C. Figure 3B showed that the activities of both enzymes increased concomitantly with increasing temperature. The activities of both AtCYN and OsCYN were >2-fold higher at 34°C than that at 19°C. Thus, the effect of temperature on the activities of AtCYN and OsCYN was similar, whereas the effect of pH differed.

### Involvement of ATCYN and OsCYN in cyanate decomposition in vivo

**Figure 4:**
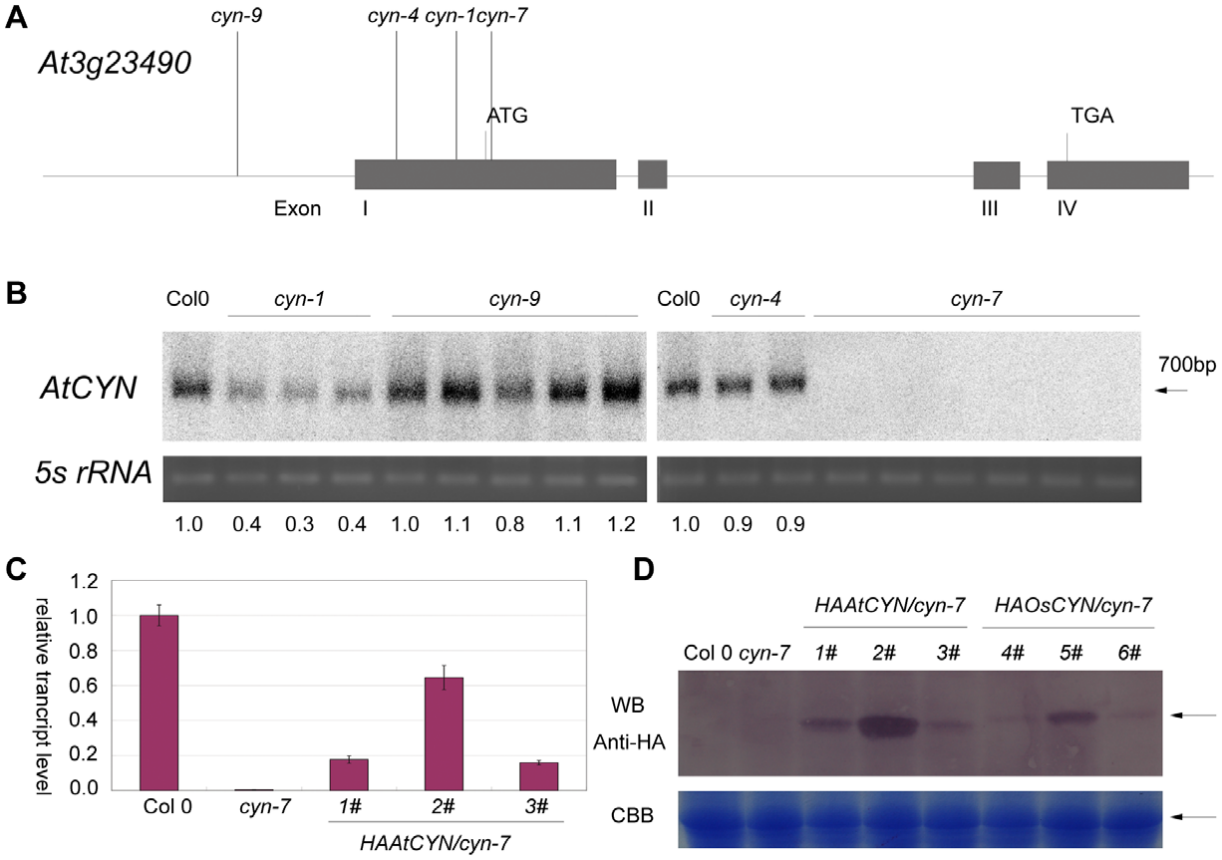
Identification of *Atcyn* mutant plants and transgenic plants. (A) Schematic diagram of the *AtCYN* gene and the T-DNA insertion positions. Gray boxes represent exons. Lines above the gene indicate T-DNA insertion positions. Accession numbers of the *Atcyn* mutant lines was listed in Table 4. (B) Northern blot analysis of *AtCYN* transcripts in *cyn* mutants. Total RNA was isolated from 14-day-old seedlings. Different homozygous individuals identified were analyzed in different lanes. Blot signals (indicated by the arrow) were quantified with ImageJ version 1.4 software and the values are presented below each lane. The 5S rRNA was visualized with ultraviolet light and was the loading control. (C) Quantitative RT-PCR analysis of *AtCYN* transcripts in Col 0, *cyn7* and transgenic plants. Error bars represent the standard deviation of three biological replicates. (D) Semi-quantitative analysis of HA:AtCYN and HA:OsCYN in transgenic plants using western blotting (WB). Blot signals (indicated by the arrow) were quantified with ImageJ and the values are presented below the lanes. The large subunit of Rubisco was visualised by Coomassie Brilliant Blue (CBB) and was the total protein loading control (indicated by the arrow).

To study the function of cyanases in plants, we obtained four T-DNA insertion mutants of *Arabidopsis thaliana* from the Arabidopsis Biological Resource Center (Figure 4A), but we were unable to obtain *cyn* mutants of *Oryza sativa* for study. *AtCYN* transcripts were detected in the mutant plants (Figure 4B). In the *cyn-1* line, the transcript level was reduced to 30–40% of that in Col 0. *AtCYN* transcription was not detected in the *cyn-7* line. We also generated transgenic lines CaMV35S:HA:AtCYN/*cyn-7* (1#, 2# and 3#) and CaMV35S:HA:OsCYN/*cyn-7*(4#, 5# and 6#) in the *cyn-7* mutant background with CYN cDNAs from Arabidopsis and rice. *AtCYN* transcripts in three independent transgenic CaMV35S:HA:AtCYN/*cyn7* lines (1#, 2# and 3#) were 15–65% the level of Col 0 (Figure 4C). As shown in Figure 4D, HA:AtCYN and HA:OsCYN proteins were detected in these transgenic lines.

**Figure 5:**
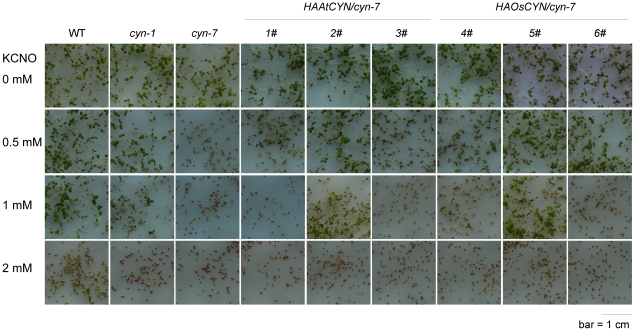
Decomposition of cyanate by AtCYN and OsCYN *in vivo*. Seeds were plated on MS medium containing either 0 mM, 0.5 mM, 1 mM or 2 mM KCNO. Plates were incubated at 4°C for 3 days and then transferred to a growth chamber for 7 days.

We treated wild-type and mutant plants with four concentrations of potassium cyanate (KCNO; Figure 5). The wild-type seeds germinated and grew normally at 0.5 mM and 1 mM KCNO but showed a slow growth at 2 mM KCNO. The knock-out mutant *cyn-7* seeds did not germinate at 1 mM and 2 mM KCNO, and grew very slowly at 0.5 mM KCNO. The knock-down mutant *cyn-1* seeds germinated at concentrations less than 1 mM KCNO, but seedlings grew more slowly than Col 0 seedlings. This experiment demonstrated that cyanate treatment inhibited germination and early seedling growth of *Arabidopsis*, and that wild-type plants showed resistance to cyanate stress. In *AtCYN* knock-down and knock-out mutant plants, the resistance to cyanate stress was weaker or lost completely.

We also treated transgenic plants with KCNO. The molecular complementation experiment was undertaken to determine whether the *cyn-7* mutant could be rescued by constitutive expression of *HA:AtCYN* and *HA:OsCYN*. As Figure 5 shows, the resistance to cyanate stress of the transgenic lines was intermediate to that of the wild-type and the knock-out mutant *cyn-7*. Lines 2# and 5#, for which the cyanase expression levels were higher than those of other lines (Figure 4D), showed higher resistance to cyanate. In particular, line 2# showed almost identical resistance as the *cyn-1* mutant, which corresponded with similar *AtCYN* transcript levels between the two lines (Figure 4B, C). The complementation experiment confirmed that cyanase contributes to *Arabidopsis* resistance to cyanate stress and provided direct evidence that plant cyanases decompose cyanate *in vivo*.

### Structural properties of AtCYN and OsCYN

**Figure 6:**
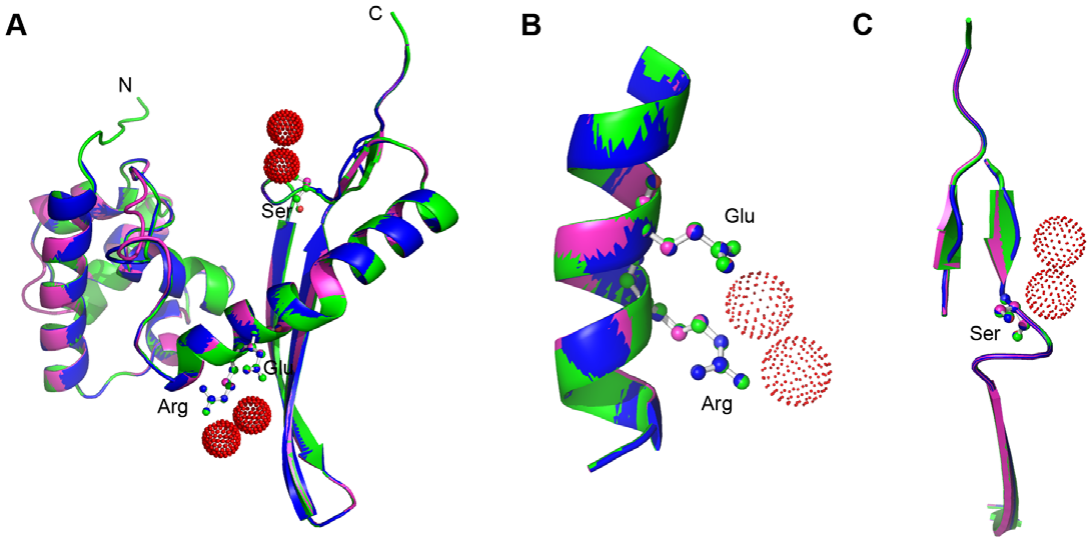
Homology modelling of AtCYN and OsCYN. (A) The predicted structures of AtCYN (blue) and OsCYN (magentas) were similar to the crystal structure of the EcCYN monomer (green). Ball-and-stick figures represent the conserved catalytic residues Arg96, Glu99 (B) and Ser122 of the EcCYN (C). Red dots indicate chloride ions.

The active *E. coli* cyanase is a homodecamer of 5 dimers comprising 17 kDa subunits, and has 3 catalytic residues Arg96, Glu99 and Ser122 [8]. To study the structural properties of AtCYN and OsCYN, we performed homology modelling using the SWISS-MODEL service. As expected, the program selected the crystal structure of *E. coli* cyanase monomer (chain J, PDB code 1dw9J) as a template. As Figure 6A shows, the backbones of the predicted structures of AtCYN and OsCYN were both similar to the crystal structure of the EcCYN monomer. Although the predicted secondary structure near the Ser at the C-terminus of ACYN was different from that of EcCYN, the positions of the three conserved catalytic residues Arg, Glu and Ser seem unchanged (Figure 6B, C). This suggests that AtCYN and OsCYN have similar monomer structures. Therefore, similarity of the quaternary structures and catalytic sites was expected.

**Figure 7:**
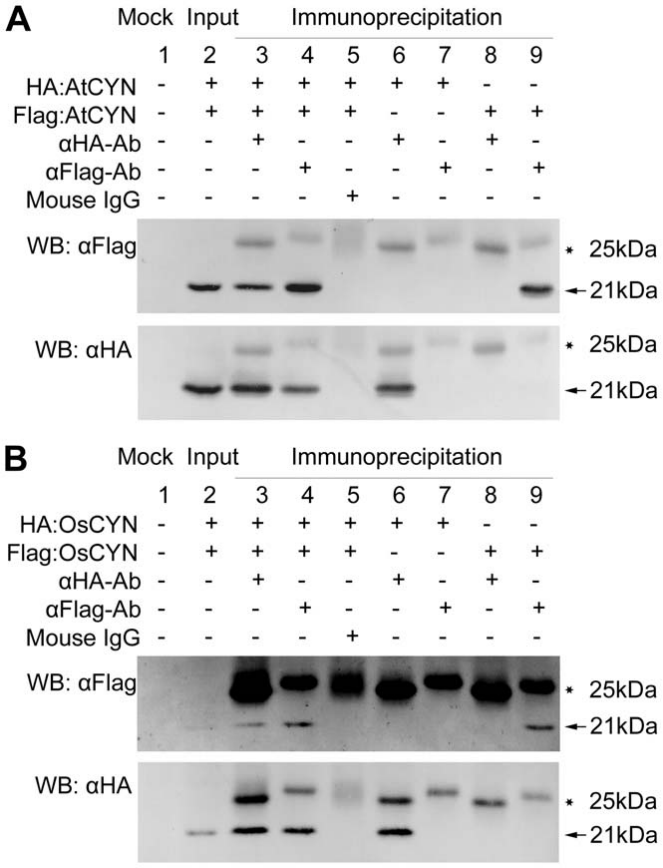
Coimmunoprecipitation assay demonstrating self-interaction of AtCYN (A) and OsCYN (B). Although an extremely low amount of OsCYN proteins was detected in the input samples, an identical pattern was shown by OsCYN in the assay. Lane 1: Mock; Lane 2: HA:CYN and FLAG:CYN were detected in the input samples; Lane 3: when anti-HA antibody was added, FLAG:CYN immunoprecipitated with HA:CYN (Lanes 6&8 were controls); Lane 4: when anti-FLAG antibody was added, HA:CYN immunoprecipitated with FLAG:CYN (Lanes 7&9 were controls); Lane 5: Native mouse IgG was used as negative control of antibodies. Bands of HA:CYN and Flag:CYN are indicated with arrows and bands of mouse IgG are indicated with stars.

We performed coimmunoprecipitation experiments to identify the self-interaction of AtCYN and OsCYN *in vivo.* Using an *Agrobacterium* infiltration assay, we transiently expressed HA-tagged and Flag-tagged CYN (AtCYN and OsCYN) in *N. benthamiana*. As shown in the upper image in Figure 7A, Flag:AtCYN coimmunoprecipitated with HA:AtCYN, and in the reciprocal experiment shown in the lower image HA:AtCYN coimmunoprecipitated with Flag:AtCYN. An identical pattern was shown by OsCYN (Figure 7B). Thus, the self-interaction of AtCYN and OsCYN was demonstrated. This indicates that either AtCYN or OsCYN could form a dimer or polymer *in vivo*.

**Figure 8:**
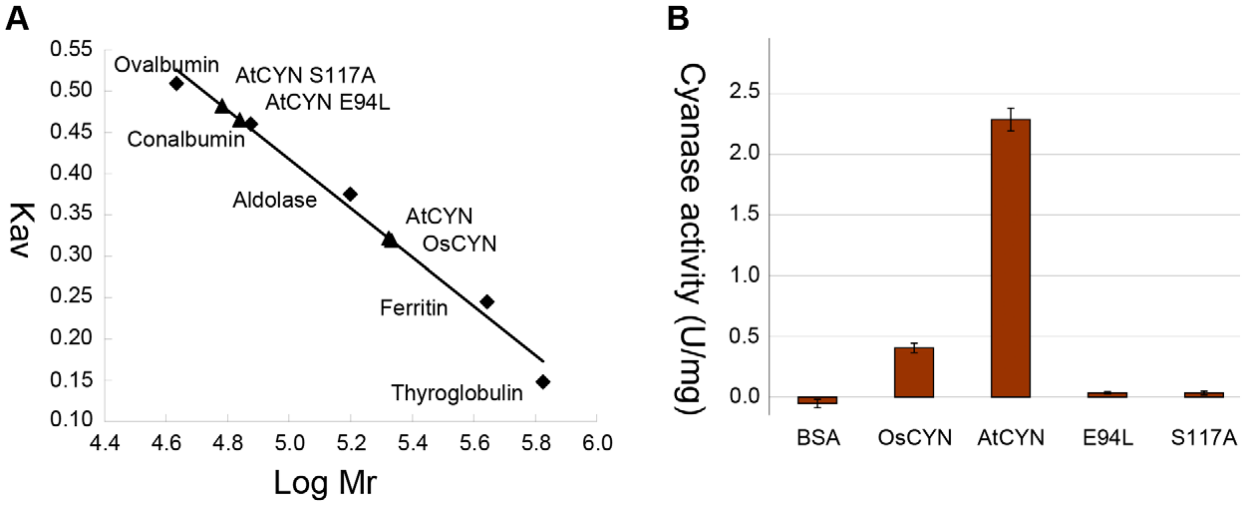
Gel filtration and cyanase activities of His-tagged AtCYN, OsCYN and AtCYN mutants (E94L and S117A). (A) Gel filtration. High Molecular Weight (HMW) Standard: Thyroglobulin, 669 kDa; Ferritin, 440 kDa; Aldolase, 158 kDa; Conalbumin, 75 kDa and Ovalbumin, 43 kDa. And calculated molecular weight: AtCYN, 210.74 kDa; OsCYN, 211.72 kDa; AtCYN-E94L, 71.64 kDa and AtCYN-S117A, 62.76 kDa. (B) Cyanase activities of His-tagged AtCYN, OsCYN and AtCYN mutants (E94L and S117A) at pH 7.7 and 27°C.

Using the gel filtration assay, we calculated the molecular weights of His-tagged recombinant CYN proteins. His:AtCYN was 210.74 kDa with a 21.9 kDa monomer, and His:OsCYN was 211.72 kDa with a 22.0 kDa monomer (Figure 8A). This demonstrates that AtCYN and OsCYN are homodecamers *in vitro*.

We constructed two His-tagged AtCYN mutants each with a single residue change: AtCYN-E94L, in which Glu94 was replaced with Leu to break the proposed salt bridge, and AtCYN-S117A, in which Ser117 was replaced with Ala. The calculated molecular weights of monomers for the both His-tagged mutant proteins were 21.9 kDa. However, gel filtration showed the molecular weight of AtCYN-E94L to be 71.64 kDa and that of AtCYN-S117A was 62.76 kDa (Figure 8A). The mutants could form trimers or a mixture of polymers but not decamers. The cyanase activities of the mutants E94L and S117A was 0.038 U·mg^–1^ and 0.040 U·mg^–1^, respectively (Figure 8B). Compared with the cyanase activity of AtCYN (2.286 U·mg^–1^), the mutants lost cyanase activity. These data demonstrate that these conserved residues are not only catalytic residues in AtCYN but also contribute to the stability of AtCYN homodecamers.

### Transcriptional regulation and expression pattern of AtCYN

**Figure 9:**
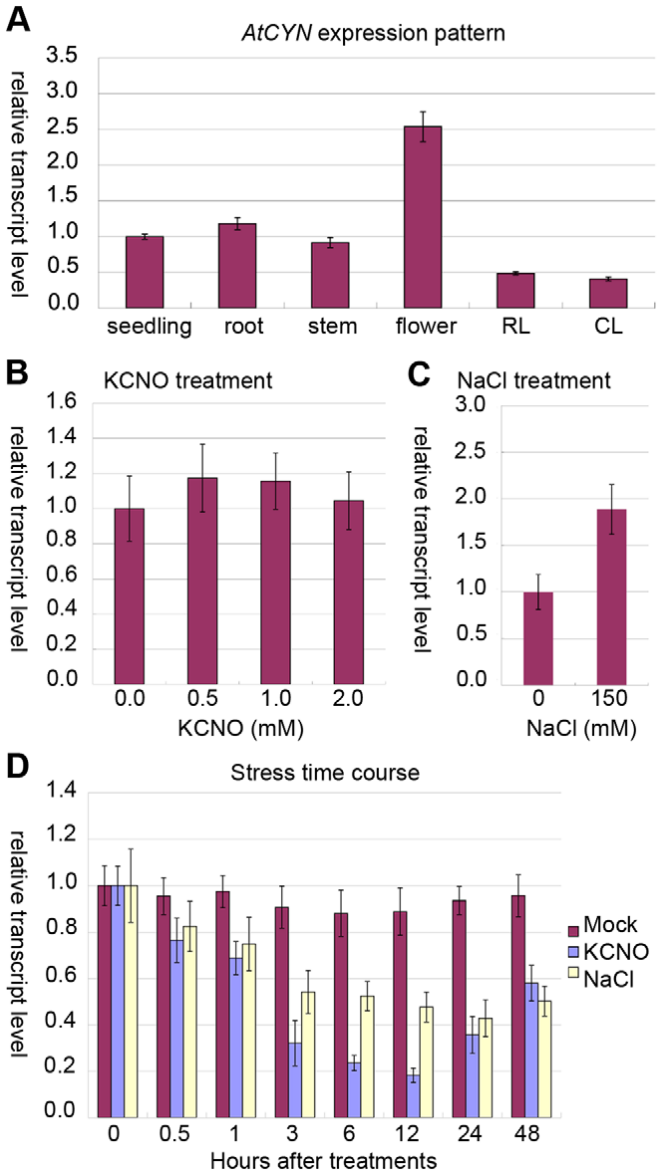
Quantitative RT-PCR analysis of *AtCYN* transcription. (A) The *AtCYN* transcript levels in different plant organs. RL (rosette leaves), CL (cauline leaves); (B) and (C) Samples are the 7-day-old seedlings which grew in the MS medium containing KCNO or NaCl. The *AtCYN* transcript levels in response to treatment with three KCNO concentrations (B) and in response to treatment with 150 mM NaCl (C); (D) The 7-day-old seedlings from the standard MS medium were transferred to the MS medium containing 1 mM KCNO or 150 mM NaCl, and the samples were harvested at different time points. Error bars represent the standard deviation of three biological replicates.

The *AtCYN* expression pattern in different *Arabidopsis* organs was examined (Figure 9A). The *AtCYN* transcript level was highest in the flower, which was nearly 3-fold higher than that in the seedling, root or stem. In rosette leaves and cauline leaves, *AtCYN* transcripts accumulated to a level about half of that in the seedling.

In the previous studies, exogenous cyanate induces the transcript level of cyanase in microbes [14], [21], [27]. And the expression level of cyanase in *Suaeda aegyptiaca* increases under salt stress [26]. The 7-day-old seedlings grew in the MS medium containing KCNO or NaCl were harvested. The *AtCYN* transcript level in the seedlings treated with three concentrations of KCNO showed no significant difference compared to that in untreated plants (Figure 9B). However, the *AtCYN* transcripts were induced by salt treatment (Figure 9C). We transferred the 7-day-old seedlings from the standard MS medium to the MS medium containing KCNO or NaCl, and harvested the samples at different time points. It was unexpected that the *AtCYN* transcripts decreased during the first 12 h under both treatments and increased thereafter (Figure 9D). The *AtCYN* transcripts were down regulated in the early stage of the treatment. But the continuous salinity stress induced *AtCYN* transcripts, while the continuous KCNO stress did not. These data suggested that a complex regulatory mechanism could control the expression of *AtCYN*.

## Discussion

To broaden our understanding of cyanases in general, we characterised cyanases from the model plants *Arabidopsis thaliana* and *Oryza sativa*. The activities of heterologously expressed AtCYN and OsCYN were measured *in vitro*, and Km values for both enzymes were calculated (Table 2). However, the activities of endogenously expressed plant cyanases were not determined directly. We isolated *cyn* mutant Arabidopsis and transformed 35S:HA:AtCYN and 35S:HA:OsCYN constructs into *cyn*-7 mutant plants. In the knock-out mutant plants, seed germination and early seedling growth were inhibited by KCNO presenting in the culture medium. However, all plants containing AtCYN or OsCYN showed cyanate resistance, and there was a positive correlation between resistance and cyanase expression levels. This indicates that plant cyanases contribute to cyanate tolerance and are involved in decomposition of cyanate in the plant.

BLAST-P searches of the NCBI databases indicated that putative cyanases are produced in some plants (Table 1). Amino acid sequences of these plant cyanases were highly conserved as are the known cyanases in prokaryotes and fungi (Figure 1). Reconstruction of phylogenetic relationships among the known cyanases (Figure 2) provided evidence for a common evolutionary origin for plant cyanases. It is suggested that the conserved cyanases are derived from an ancient gene, which makes it possible to study inter- and intra-specific relationships using cyanase as a phylogenetic marker [28]. The high resolution crystal structure of *E. coli* cyanase explains the structural and kinetic properties of the enzyme: the active enzyme is a homodecamer composed of five inactive dimmers, and catalytic residues were identified [8]. In our study, homology modelling showed the monomer structures of AtCYN and OsCYN were similar to that of EcCYN (Figure 6). And the similar active homodecamer of plant cyanases was confirmed (Figure 7 and 8). Analysis of AtCYN mutants E94L and S117A confirmed the conserved catalytic residues, and indicated that not only the glutamate but also the serine contributes to the formation of the active homodecamer (Figure 8), which was not mentioned in the previous studies of EcCYN. And the difference may be explained by the different structure near the serine (Figure 6C).

The cyanases shared higher sequence identity and some similar properties, but we also found different properties between the both plant cyanases. Although the assay conditions differ in pH and concentration of substrate, *Km* NaHCO_3_ values of both plant cyanases (0.79 mM for AtCYN and 0.63 mM for OsCYN) are approximate to that of the characterized PpCYN (CynS of *P. pseudoalcaligenes*, 0.67 mM) and SmCYN (CYN1 of *Sordaria macrospore*, 0.83 mM) [14], [21]. However, the characterized cyanases differ in *Km* KCNO values (0.94 mM for AtCYN, 7.38 mM for OsCYN, 0.6 mM for EcCYN, 2.40 mM for PpCYN and 0.58 mM for SmCYN) [12], [14], [21]. These data suggested these cyanases have similar binding affinity to bicarbonate but different binding affinity to cyanate, although the substrates bicarbonate and cyanate share same binding sites of the enzymes [8]. The characterised cyanases are sensitive to the change in pH [12], [14], [21]. AtCYN was pH-sensitive, and its optimum pH value was 7.7, which is similar to EcCYN and SmCYN [12], [21]. Differently, AtCYN lost enzyme activity at low pH, which suggested the enzyme was destabilized in acidic environment. However, OsCYN was less pH-sensitive, and its optimum pH value was 5.7, while that of the characterised enzymes in *E. coli, P. pseudoalcaligenes* and *S. macrospore* is near neutral or alkaline [12], [14], [21]. The two plant cyanases with high sequence-identity were differed so far at low pH. The environment temperature changes during the life cycle of plants. The activity of AtCYN and OsCYN increased concomitantly with the increasing temperature. In our study, the cyanase activity of heterologously expressed AtCYN is 5-fold higher than OsCYN. On the contrary, in the complementary experiment, although the amount of the enzyme in the line 2# was more than that of the line 5# (Figure 4D), the line 5# showed higher resistance to cyanate (Figure 5), which suggested that OsCYN showed higher activity than AtCYN in Arabidopsis. We suspect that the differences between the protein translation, folding and modification in plants and in *E. coli* caused the opposite results in the two experiments.

Cyanate in the soil may come from solutions containing urea or cyanide in nature, and human industry released more cyanate into the environment [1], [2], [3], [4]. Researchers have discovered some details of resistance and utilization of exogenous cyanate in microbes [17], [23], [24], and people have used cyanate to control crabgrass for many years [29], however, what happened between plants and exogenous cyanate is not clear. In our study, the model plant Arabidopsis was treated with exogenous cyanate. And the both plant cyanases help Arabidopsis resist exogenous cyanate. In plants, one possible endogenous source of cyanate is involved in metabolism pathway of cyanide compounds[23], such as cyanide [30] and thiocyanate [31]. Cyanide is a co-product of the degradation of ACC (1-Aminocyclopropane-carboxylic acid) into ethylene [32]; and both cyanide and ethylene play roles in physiological processes in the life cycle of plants and in responses to biotic and abiotic stress [33], [34], [35], [36]. Another possible endogenous source of cyanate is the dissociation of carbamoyl phosphate [37], which servers as a precursor for arginine and pyrimidine synthesis [38], [39]. In previous studies in microbes, cyanases are involved in metabolism pathway of cyanide, thiocyanate and carbamoyl phosphate [21], [40], [41]. Cyanase may also play roles in these processes in plants. Furthermore, cyanase is supposed to be involved in physiological processes of ethylene, arginine and pyrimidine.

We found exogenous cyanate did not induce *AtCYN* transcription. But in the previous studies in microbes, the transcriptional level of cyanase is induced by exogenous cyanate [14], [21], [27]. However, the transcriptional regulation in plants differs from that in microbes. It was reported that expression of cyanase from *Suaeda aegyptiaca* can be induced by salt stress, and analysis of upstream noncoding sequence revealed an ethylene-responsive element (ATTTAAAA) in *Arabidopsis thaliana* and *Oryza sativa* [26]. In our study, salt stress also led to accumulation of *AtCYN* RNA. In particular, *AtCYN* transcripts decreased during the first 12 h of salt treatment and increased thereafter, which was similar to the pattern of *AtCYN* transcription under KCNO stress. And *AtCYN* transcripts were higher in the flower than in other organs of Arabidopsis. Ethylene regulates many aspects of the plant life cycle, such as seed germination, flower development and fruit ripening [42]. Ethylene has also been regarded as a stress hormone [43]. Ethylene signaling is involved in plant responses to abiotic stress, such as salt, cold and osmotic stress, and ethylene biosynthesis is regulated by stress factors [44], [45], [46], [47], [48]. Therefore, the ethylene-responsive elements suggested ethylene signaling could be involved in the regulation of cyanase in plants, and plant cyanase could play roles in a variety of developmental processes and stress responses.

The biophysical roles of cyanases have been investigated in certain bacteria [17], [24] but remain unclear. With regard to more complicated organisms such as plants, the present study contributes to the characterisation of two plant cyanases, but further studies are needed to understand the regulation and the exact roles of cyanase in plants.

## Materials and Methods

### Plant materials and growth conditions

*Arabidopsis thaliana* lines of the Col 0 background and *O. sativa* L. cv. Zhonghua 11 were used. The T-DNA insertion mutant lines were obtained from the Arabidopsis Biological Resources Center, Ohio State University. Seeds were plated on Murashige and Skoog (MS) medium supplemented with 0.8% agar and 1% sucrose, incubated at 4°C for 3 days then transferred to a growth chamber with a 14 h light/22°C and 10 h dark/18°C regime. *Nicotiana benthamiana* plants were grown in a growth chamber under a 12 h light/25°C and 12 h dark/18°C regime.

### Sequence analysis and homology modelling

Protein sequence data for cyanase genes from other organisms were obtained by BLAST-P searches of the National Center for Biotechnology Information (NCBI) Entrez databases [49]. Multiple amino acid sequence alignments were performed using Clustal X software [50]. An unrooted phylogenetic tree was constructed using the neighbour-joining method with Clustal X and visualised using TreeView [51].

The homology structure modelling of AtCYN and OsCYN was performed using Swiss Model Service [52] based on the structure of *E. coli* cyanase (PDB code 1dw9J) as a template. The resulting models were analysed using PyMOL program (Version 1.1).

### cDNA cloning and vector construction

Total RNA was isolated from various tissues of *A. thaliana* and *O. sativa* using TRIzol® Reagent (Invitrogen) according to the manufacturer's instructions. Reverse transcription was performed using Superscript II reverse transcriptase (Invitrogen) and the primers AtCYN 3′ and OsCYN 3′ (Table 3). The primer pairs AtCYN 5′/3′ and OsCYN 5′/3′ were used for reverse transcription PCR. *AtCYN* and *OsCYN* cDNAs were cloned into pBluescript II SK(-) at the *EcoR V* site to obtain the entry vectors pBS-AtCYN and pBS-OsCYN. The *BamH I* and *Xho I* sites were used to clone *AtCYN* and *OsCYN* from the entry vectors into the pET15b expression vector (Novagen), resulting in pET-AtCYN and pET-OsCYN. The mutants E94L and S117A of pET-AtCYN were constructed with the KOD-Plus Mutagenesis Kit (Toyobo), using the mutagenesis primer pairs E94L FP/RP and S117A FP/RP (Table 3). The pET-CYN constructs were introduced into *E. coli* BL21 (DE3) cells (Stratagene). *Spe I* and *Cla I* were used to clone *AtCYN* and *OsCYN* into the pRTL2HA and pRTL2Flag vectors. The *HA:CYN* and *Flag:CYN* fragments were digested with *Sph I* and blunted with T4 DNA polymerase, then inserted into the binary vector pWM101 at the *Sma I* site. Expression of *HA:CYN* and *Flag:CYN* was under control of the CaMV 35S promoter. The pWM-CYN constructs were introduced into *Agrobacterium tumefaciens* strain GV3101 by electroporation.

**Table 3:** List of primers used in this study.

Primer name	Primer sequence
AtCYN 5′	5′-ATGGAAGCGGCGAAGAAACAGAGT-3′
AtCYN 3′	5′-TCATTCGCTTGTACCTCCCTTGAG-3′
OsCYN 5′	5′-ATGGAGGGCGGCGGCGGGGAGAGG-3′
OsCYN 3′	5′-TCAAGATGTCTTGCGGGTCAGCCT-3′
E94L FP	5′-CTAGCAGTGATGCATTTTGGTGAGAG-3′
E94L RP	5′-ATTCAACCTGTAGATAGTGGGTTCTTGG-3′
S117A FP	5′-GCGGCGATAGATTTCTATTGCTCTG-3′
S117A RP	5′-CATGATGCCATCTCCAAAATCTTC-3′
LBb1	5′-GCGTGGACCGCTTGCTGCAACT-3′
LP1	5′-TCCGATTAGTTCATCGGTCAG-3′
LP2	5′-TTCGATTTTGTTCATAGTTCATGAC-3′
RP1	5′-AGGCCGGCGCTTACTATATAC-3′
RP2	5′-ACCAAAATGCATCACTGCTTC-3′
RP3	5′-TCCGATTAGTTCATCGGTCAG-3′
AtCYN P5	5′-GCGGAGACAGGTCTAACCAAC-3′
AtCYN P6	5′-ACTTTCCATCAAGCGTCACAA-3′
AtEIF4AF	5′- GCGCATCCTCCAAGCTGGTGTCC-3′
AtEIF4AR	5′-GGTGGAAGAAGCTGGAATATGTCAT-3′

### Protein preparation and gel filtration assay

*Escherichia coli* BL21 (DE3) cells containing the pET-CYN constructs were cultured in LB medium and His-tagged recombinant proteins were induced by adding 0.5 mM isopropyl β-D-thiogalactoside for 1–3 hours at 37°C. Cells were harvested and sonicated at 4°C. First, affinity chromatography was performed to purify the His-tagged proteins using Ni^2+^ chelating columns, and then the samples were loaded onto a Superdex 200 10/300 GL column for gel filtration chromatography.

### Cyanase activity assay

Cyanase activity *in vitro* was determined by monitoring ammonia formation in the reaction medium as described previously [12]. The standard assay solution was comprised of 50 mM potassium phosphate buffer (pH 7.7) containing 3 mM sodium bicarbonate and 2 mM potassium cyanate. The reaction was started by the addition of the cyanase and terminated by the addition of equal volumes of Nessler reagent after 1–10 min at 27°C. The amount of ammonia formed was determined by monitoring the absorbance at 420 nm within 10 min of adding Nessler reagent. One unit of cyanase was defined as the amount of enzyme that catalyses the formation of 1 µmol NH_3_ min^−1^ under the assay conditions. To allow for the influence of temperature, the reaction was assayed at 19°C, 27°C and 34°C. To investigate the influence of pH, an assay solution pH range between 4 and 9 was used.

### Coimmunoprecipitation assay

*Agrobacterium* strains carrying the pWM-HA:CYN and pWM-Flag:CYN constructs were cultured in LB medium at 28°C until OD_600_ >0.8. The cells were harvested and resuspended in infiltration medium (10 mM MgCl_2_, 10 mM MES pH 5.7 and 20 µM acetosyringone) to OD_600_ 0.8. Equal volumes of cell suspensions for the two strains were mixed and injected into leaves of *N. benthamiana.* After transient expression for 24–48 h, the leaves were harvested and ground to a powder in liquid nitrogen. The powder was thawed in two volumes of immunoprecipitation (IP) buffer (50 mM Tris–HCl pH 7.5, 150 mM NaCl, 0.1% Nonidet P-40, and 1× complete protease inhibitor cocktail; Roche) and centrifuged at 12,000×g for 10 min at 4°C. After centrifugation, 1 ml of supernatant was incubated with 50 µl of protein G Sepharose-4 fast flow beads (Amersham) and 0.5 µg of the indicated monoclonal antibody for 3 h at 4°C. After incubation, the immunocomplexes were washed four times with 1 ml IP buffer. The pellet samples were separated by SDS-PAGE and analysed by western blotting. The following antibodies were used: Mouse anti-FLAG antibody (clone M2, F3165, Sigma), Mouse anti-HA antibody (clone HA-7, H9658, Sigma), Goat anti-Mouse IgG (H+L) (Alkaline phosphatase conjugate, S3721, Promega) and Native mouse IgG (401111, Merck).

### Identification of T-DNA Insertion and Plant Transformation

The *Atcyn* mutant plants were genotyped by PCR using the specific primers LBa1, LP1, LP2, RP1, RP2 and RP3 (Table 3) designed with SALK (http://signal.salk.edu/tdnaprimers.2.html). Accession numbers of the *Atcyn* mutant lines was listed in Table 4. The floral dip method [53] was used to transform the pWM-CYN constructs into *cyn-7* mutant plants.

**Table 4:** Accession numbers of the *Atcyn* mutant lines.

*Atcyn* mutant line	Accession number
*cyn-1*	SALK_026383.12.70.x
*cyn-4*	SALK_064817.47.60.x
*cyn-7*	SALK_108492.22.55.x
*cyn-9*	SALK_127670.27.75.x

### Northern blotting and quantitative RT-PCR analysis

Total RNA isolation and reverse transcription were performed as described above. The *AtCYN* segment was amplified using the primer pair AtCYN 5′/3′, and labelled with α-^32^P-dCTP using the Prime-a-Gene Labeling System (Promega). Hybridization was performed in 50% formamide, 5× SSC, 5× Denhardt's solution and 1% SDS with 100 mg/ml sperm DNA at 42°C. After overnight incubation, the blot membranes were washed twice in 2× SSC and 0.1% SDS for 10 min at room temperature and three times in 0.2× SSC and 0.1% SDS for 30 min at 42°C. Autoradiography of the blot membranes was carried out using the Cyclone phosphor imaging system (Packard Instruments).

Real-time PCR was performed using the SYBR® Green PCR Master Mix (Toyobo) with a DNA Engine Opticon TM 2 system (Bio-Rad). Each reaction was run in three technical replicate with at least three independent biological replicates. The primers used to amplify *AtCYN* were AtCYN P5/P6 and those used to amplify the internal control *AtEIF4A* were AtEIF4AF/R [54](Table 3). Relative transcript levels of *AtCYN* (RE) were calculated using the following equation: RE = (*E _AtCYN_*) ^ΔCt *AtCYN*(control-sample)^/(*E _AtEIF4A_*) ^ΔCt *AtEIF4A* (control-sample)^ [55].

*E _AtCYN_* = 1.62 and *E _AtEIF4A_* = 1.78.

## References

[1] Sancho JFB, Bellón F (2005). Developments of an alternative technology to remove cyanide from mining wastewater.. I9th International Mine Water Congress.

[2] Dirnhuber P, Schutz F (1948). The isomeric transformation of urea into ammonium cyanate in aqueous solutions.. Biochemical Journal.

[3] Malhotra S, Pandit M, Kapoor JC, Tyagi DK (2005). Photo-oxidation of cyanide in aqueous solution by the UV/H2O2 process.. Journal of Chemical Technology and Biotechnology.

[4] Rader WS, Solujic L, Milosavljevic EB, Hendrix JL, Nelson JH (1995). Photocatalytic detoxification of cyanide and metal cyano-species from precious-metal mill effluents.. Environmental Pollution.

[5] Kraus LM, Kraus AP (2001). Carbamoylation of amino acids and proteins in uremia.. Kidney International.

[6] Stark GR (1965). Reactions of cyanate with functional groups of proteins. 3. Reactions with amino and carboxyl groups.. Biochemistry.

[7] Johnson WV, Anderson PM (1987). Bicarbonate is a recycling substrate for cyanase.. Journal of Biological Chemistry.

[8] Walsh MA, Otwinowski Z, Perrakis A, Anderson PM, Joachimiak A (2000). Structure of cyanase reveals that a novel dimeric and decameric arrangement of subunits is required for formation of the enzyme active site.. Structure.

[9] Sung YC, Parsell D, Anderson PM, Fuchs JA (1987). Identification, mapping, and cloning of the gene encoding cyanase in Escherichia coli K-12.. Journal of Bacteriology.

[10] Anderson PM, Little RM (1986). Kinetic properties of cyanase.. Biochemistry.

[11] Chin CCQ, Anderson PM, Wold F (1983). The amino acid sequence of Escherichia coli cyanase.. Journal of Biological Chemistry.

[12] Anderson PM (1980). Purification and properties of the inducible enzyme cyanase.. Biochemistry.

[13] Taussig A (1960). The synthesis of the induced enzyme, cyanase, in E. coli.. Biochimica Et Biophysica Acta.

[14] Luque-Almagro VM, Huertas MJ, Saez LP, Luque-Romero MM, Moreno-Vivian C (2008). Characterization of the Pseudomonas pseudoalcaligenes CECT5344 cyanase, an enzyme that is not essential for cyanide assimilation.. Applied and Environmental Microbiology.

[15] Wood AP, Kelly DP, McDonald IR, Jordan SL, Morgan TD (1998). A novel pink-pigmented facultative methylotroph, Methylobacterium thiocyanatum sp. nov., capable of growth on thiocyanate or cyanate as sole nitrogen sources.. Archives of Microbiology.

[16] Deckert G, Warren PV, Gaasterland T, Young WG, Lenox AL (1998). The complete genome of the hyperthermophilic bacterium Aquifex aeolicus.. Nature.

[17] Kunz DA, Nagappan O (1989). Cyanase-mediated utilization of cyanate in Pseudomonas fluorescens NCIB 11764.. Applied and Environmental Microbiology.

[18] Dorr PK, Knowles CJ (1989). Cyanide oxygenase and cyanase activities of Pseudomonas fluorescens NCIMB11764.. Fems Microbiology Letters.

[19] Harano Y, Suzuki I, Maeda SI, Kaneko T, Tabata S (1997). Identification and nitrogen regulation of the cyanase gene from the cyanobacteria Synechocystis sp. strain PCC 6803 and Synechococcus sp. strain PCC 7942.. Journal of Bacteriology.

[20] Miller AG, Espie GS (1994). Photosynthetic metabolism of cyanate by the cyanobacterium Synechococcus UTEX 625.. Archives of Microbiology.

[21] Elleuche S, Poggeler S (2008). A cyanase is transcriptionally regulated by arginine and involved in cyanate decomposition in Sordaria macrospora.. Fungal Genetics and Biology.

[22] Aichi M, Nishida I, Omata T (1998). Molecular cloning and characterization of a cDNA encoding cyanase from Arabidopsis thaliana.. Plant and Cell Physiology Supplement.

[23] Ebbs S (2004). Biological degradation of cyanide compounds.. Current Opinion in Biotechnology.

[24] Espie GS, Jalali F, Tong T, Zacal NJ, So AKC (2007). Involvement of the cynABDS operon and the CO2-concentrating mechanism in the light-dependent transport and metabolism of cyanate by cyanobacteria.. Journal of Bacteriology.

[25] Gyorgyey J, Vaubert D, Jimenez-Zurdo JI, Charon C, Troussard L (2000). Analysis of Medicago truncatula nodule expressed sequence tags.. Molecular Plant-Microbe Interactions.

[26] Askari H, Edqvist J, Hajheidari M, Kafi M, Salekdeh GH (2006). Effects of salinity levels on proteome of Suaeda aegyptiaca leaves.. Proteomics.

[27] Sung YC, Fuchs JA (1992). The Escherichia coli K-12 cyn operon is positively regulated by a member of the lysR family.. Journal of Bacteriology.

[28] Wu Z, Snabel V, Pozio E, Hurnikova Z, Nareaho A (2007). Genetic relationships among Trichinella pseudospiralis isolates from Australian, Nearctic, and Palearctic regions.. Parasitology Research.

[29] Zimmermani WE (1950). Experiments with potassium cyanate to control crabgrass in turf.. Greenkeeper's Reporte.

[30] Kunz DA, Fernandez RF, Parab P (2001). Evidence that bacterial cyanide oxygenase is a pterin-dependent hydroxylase.. Biochemical and Biophysical Research Communications.

[31] Sorokin DY, Tourova TP, Lysenko AM, Kuenen JG (2001). Microbial thiocyanate utilization under highly alkaline conditions.. Applied and Environmental Microbiology.

[32] Yip WK, Yang SF (1988). Cyanide metabolism in relation to ethylene production in plant tissues.. Plant Physiology.

[33] Siegien I, Bogatek R (2006). Cyanide action in plants - from toxic to regulatory.. Acta Physiologiae Plantarum.

[34] Chen YF, Etheridge N, Schaller GE (2005). Ethylene signal transduction.. Annals of Botany.

[35] Penninckx IAMA, Thomma BPHJ, Buchala A, Metraux JP, Broekaert WF (1998). Concomitant activation of jasmonate and ethylene response pathways is required for induction of a plant defensin gene in Arabidopsis.. Plant Cell.

[36] O'Donnell PJ, Calvert C, Atzorn R, Wasternack C, Leyser HMO (1996). Ethylene as a signal mediating the wound response of tomato plants.. Science.

[37] Lawrie AC (1979). Effect of carbamoyl phosphate on nitrogenase in Anabaena cylindrica Lemm.. Journal of Bacteriology.

[38] Potel F, Valadier MH, Ferrario-Mery S, Grandjean O, Morin H (2009). Assimilation of excess ammonium into amino acids and nitrogen translocation in Arabidopsis thaliana- roles of glutamate synthases and carbamoylphosphate synthetase in leaves.. Febs Journal.

[39] Slocum RD (2005). Genes, enzymes and regulation of arginine biosynthesis in plants.. Plant Physiology and Biochemistry.

[40] Grigor'eva NV, Kondrat'eva TF, Krasil'nikova EN, Karavaiko GI (2006). Mechanism of cyanide and thiocyanate decomposition by an association of Pseudomonas putida and Pseudomonas stutzeti strains.. Microbiology.

[41] Luque-Almagro VM, Huertas MJ, Martinez-Luque M, Moreno-Vivian C, Roldan MD (2005). Bacterial degradation of cyanide and its metal complexes under alkaline conditions.. Applied and Environmental Microbiology.

[42] Lin ZF, Zhong SL, Grierson D (2009). Recent advances in ethylene research.. Journal of Experimental Botany.

[43] Morgan PW, Drew MC (1997). Ethylene and plant responses to stress.. Physiologia Plantarum.

[44] Wang HH, Liang XL, Wan Q, Wang XM, Bi YR (2009). Ethylene and nitric oxide are involved in maintaining ion homeostasis in Arabidopsis callus under salt stress.. Planta.

[45] Cao WH, Liu J, He XJ, Mu RL, Zhou HL (2007). Modulation of ethylene responses affects plant salt-stress responses.. Plant Physiology.

[46] Achard P, Cheng H, De Grauwe L, Decat J, Schoutteten H (2006). Integration of plant responses to environmentally activated phytohormonal signals.. Science.

[47] Zhao XC, Schaller GE (2004). Effect of salt and osmotic stress upon expression of the ethylene receptor ETR1 in Arabidopsis thaliana.. Febs Letters.

[48] Zanetti ME, Terrile MC, Arce D, Godoy AV, San Segundo B (2002). Isolation and characterization of a potato cDNA corresponding to a 1-aminocyclopropane-1-carboxylate (ACC) oxidase gene differentially activated by stress.. Journal of Experimental Botany.

[49] Altschul SF, Madden TL, Schaffer AA, Zhang JH, Zhang Z (1997). Gapped BLAST and PSI-BLAST: a new generation of protein database search programs.. Nucleic Acids Research.

[50] Thompson JD, Gibson TJ, Plewniak F, Jeanmougin F, Higgins DG (1997). The CLUSTAL_X windows interface: flexible strategies for multiple sequence alignment aided by quality analysis tools.. Nucleic Acids Research.

[51] Page RDM (1996). TreeView: An application to display phylogenetic trees on personal computers.. Computer Applications in the Biosciences.

[52] Arnold K, Bordoli L, Kopp J, Schwede T (2006). The SWISS-MODEL workspace: a web-based environment for protein structure homology modelling.. Bioinformatics.

[53] Clough SJ, Bent AF (1998). Floral dip: a simplified method for Agrobacterium-mediated transformation of Arabidopsis thaliana.. Plant Journal.

[54] Hunter C, Willmann MR, Wu G, Yoshikawa M, de la Luz Gutierrez-Nava M (2006). Trans-acting siRNA-mediated repression of ETTIN and ARF4 regulates heteroblasty in Arabidopsis.. Development.

[55] Pfaffl MW (2001). A new mathematical model for relative quantification in real-time RT-PCR.. Nucleic Acids Research.

